# Magnetic-Field-Based Indoor Positioning Using Temporal Convolutional Networks

**DOI:** 10.3390/s23031514

**Published:** 2023-01-30

**Authors:** Guanglie Ouyang, Karim Abed-Meraim, Zuokun Ouyang

**Affiliations:** Laboratoire Pluridisciplinaire de Recherche en Ingénierie des Systèmes, Mécanique et Energétique, Université d’Orléans, 12 Rue de Blois, 45067 Orleans, France

**Keywords:** magnetic field, indoor positioning, temporal convolutional networks, magnetic trajectories, heterogenous smartphones

## Abstract

Traditional magnetic-field positioning methods collect magnetic-field information from each spatial point to construct a magnetic-field fingerprint database. During the positioning phase, real-time magnetic-field measurements are matched to a magnetic-field map to predict the user’s location. However, this approach requires a significant amount of time to traverse the entire magnetic-field fingerprint database and does not effectively leverage the magnetic-field sequence’s unique patterns to improve the accuracy and robustness of the positioning system. In recent years, the application of deep learning for the indoor positioning of magnetic fields has grown rapidly, especially by using the magnetic-field sequence as a time series and a trained long short-term memory (LSTM) model to predict the position, directly avoiding the time-consuming matching process. However, the training of LSTM is time-consuming, and the degradation problem occurs as the stack of layers increases. This article proposes a temporal convolutional network (TCN)-based magnetic-field positioning system that extracts magnetic-field sequence features by preprocessing them with coordinate transformation, smoothing filtering, and first-order differencing. The proposed method is seamlessly applicable to heterogeneous smartphones. The trained TCN models are compared with the LSTM and gated recurrent unit (GRU) models, showing the high accuracy and robustness of the proposed algorithm.

## 1. Introduction

In recent years, the rising demand for accurate and timely location-based services (LBSs) has attracted considerable interest from academics and the industry. Advanced positioning technology can provide better services such as indoor navigation and tracking, entertainment, location-based information retrieval, and emergency and safety applications [1,2].

Infrastructural approaches include Wi-Fi, radio frequency identification (RFID), ultrawide-band (UWB), and Bluetooth (BLE), and they require a customized infrastructure such as Wi-Fi access points (APs), beacons, sensors, and tags to sense the environment. Pedestrian dead reckoning (PDR) and magnetic-field-based location systems employ environmental signals and do not require an infrastructure [3,4,5,6].

Wi-Fi [7] has an average accuracy of 5 to 15 m. It has the advantage of widely distributed Wi-Fi APs, low access requirements, and high flexibility. However, it also has limitations such as noise and multipath distortion, radio mismatch issues, fluctuations in Wi-Fi signals, vulnerability to changes in APs, and the heterogeneity of Wi-Fi devices, and the positioning performance is severely degraded in dynamic environments. In addition, in recent times, Android has restricted the frequent scanning of Wi-Fi APs (Wi-Fi scan throttling), limiting the widespread use of Wi-Fi location methods [8].

BLE [9] has been the focus of attention for indoor positioning technologies, with an accuracy of typically 1 to 5 m. It has the advantage of a low reception range and low energy consumption. However, BLE is expensive as it requires the intensive deployment of BLE beacons to improve positioning accuracy. It also has inherent limitations in radio signal propagation, such as shadowing, signal absorption, and multipath.

UWB [10] has the advantages of high accuracy (10∼30 cm), high multipath resolution, large bandwidth, low latency, high penetration, and freedom from interference. The constraints of UWB include high infrastructural requirements, energy consumption, and user costs.

Inertial navigation [11] is advantageous due to its low cost and ease of deployment; its disadvantage is that it is restricted by the accuracy of inertial sensors, and the accumulation of drift and deviation errors.

Magnetic-field-based indoor positioning is an attractive candidate for indoor positioning solutions due to the prevalence of magnetic fields. The advantages of magnetic fields are that they are infrastructure-free, they have temporal stability, and are tolerant to moving objects. There are also some disadvantages, such as low discernibility (i.e., identical magnetic-field measurements can be found elsewhere), the heterogeneity of devices (i.e., heterogeneous smartphones have different magnetic-field measurements at the same location), and the susceptibility to interference from the presence of ferromagnetic materials in the surrounding environment [12].

The contributions of this study are summarized as follows.

- A magnetic-field-based indoor positioning system was designed. Six heterogenous smartphones, namely, iPhone 12 Mini, iPhone Xs Max, Redmi Note 7, Samsung Galaxy S20, Samsung Galaxy S9, and Oneplus 7T Pro, were used to collect magnetic-field trajectories to construct an extensive database of magnetic-field trajectories.
- Compared to traditional machine learning and the dynamic time warping (DTW) method, the proposed method does not require the traversal of the entire magnetic-field database.
- Compared to recurrent neural network (RNN) methods (e.g., long- short-term memory (LSTM) and the gated recurrent unit (GRU)), the proposed method avoids the degradation problem as the number of stack layers increases. Conventional RNNs such as LSTM/GRU are nonparallel learning systems that must complete the previous hidden state’s computation before the subsequent hidden state’s computation, whereas the temporal convolutional network (TCN) is a parallel system that requires much less training time [13].
- Magnetic-field measurements are preprocessed using magnetic-field coordinate system transformation, moving average, and first-order difference methods.
- The trained model was used to classify the magnetic-field sequences from the test set, achieving 99.80% accuracy for the three trained smartphones. For the untrained heterogeneous smartphones (Samsung Galaxy S20, Samsung Galaxy S9, and OnePlus 7T Pro), accuracies of 95.20%, 88.23%, and 84.27% were achieved, respectively. The proposed method, thus, functions well for heterogeneous devices.

The rest of the article is organized as follows: Section 2 provides a brief review of previous work on indoor positioning using magnetic fields. Section 3 and Section 4 present a preliminary analysis of magnetic-field data and a background overview of TCNs, respectively. Then, the proposed architecture, experimental setup and results, and analysis are explained in Section 5. Lastly, Section 6 concludes our work.

## 2. Related Work

Due to its simplicity and effectiveness, the fingerprinting method is widely used for indoor positioning on the basis of magnetic fields. The fingerprinting approach consists of two phases, online and offline, starting with an online phase when data from ground-truth locations are collected to create a database, followed by an offline phase when the data from the smartphone are used for positioning.

Magnetic fields are mainly used in narrow one-dimensional spaces such as corridors [14,15]. There are difficulties in using magnetic fields in wide environments [16], and it is challenging to achieve positioning using magnetic fields in old buildings with almost no metal structures [17].

The fingerprinting positioning problem can often be seen as a classification problem, and many machine-learning methods, such as *k*-nearest neighbors (*k*-NN) [18], support vector machines (SVMs) [19,20], decision trees [21], and neural networks [22,23], can be used to predict the location by classifying the fingerprinting signal. Montoliu et al. [24] proposed a bag-of-words (BoW)-based method for describing fingerprints on the basis of magnetic fields. The authors gathered 21 points in a corridor and evaluated the classification using *k*-NN, SVM, random forest, and other algorithms, demonstrating good positioning performance.

Typically, fingerprinting methods require traversing the entire magnetic-field database, which is often time-consuming. Most conventional methods treat successive sequences of magnetic fields as independent observations of each other, and positioning is achieved with point-to-point matching. However, measurements from two points at different spatial locations may show similarity in a wide environment, leading to positioning errors. Complex indoor environments often have various constraints, and as some walking trajectories are relatively fixed, it becomes feasible to use historical information about the trajectory to enhance the location estimation method [25].

In [26,27], the authors used dynamic time warping (DTW), which treats the magnetic-field trajectory as a time series. The magnetic-field trajectories’ unique pattern can overcome the low-discernibility problem of the magnetic field, but it is also very time-consuming and can cause time delays. Although magnetic-field anomalies can be used to locate users moving through narrow corridors, it remains challenging to locate users in expansive areas. Perez-Navarro et al. [27] created eight ’virtual corridors’ to simulate users’ movement upon entering this building. Using the DTW method, they obtained a dataset of 64 paths (8 paths × 2 directions × 4 smartphones) and achieved corridor-level positioning.

The works in [28,29,30] used sensor fusion methods to increase positioning accuracy, such as the fusion of pedestrian dead reckoning (PDR) with the magnetic field using Kalman filters, particle filters, or hidden Markov models. However, these methods improve positioning performance by increasing the information, and do not improve positioning methods that use only the magnetic field. Furthermore, filter-based sensor fusion requires sufficient experience to tune parameters such as the covariance matrix [26].

The use of deep-learning algorithms in indoor positioning has grown rapidly in recent years. In [31,32,33], the authors highlighted the problem of heterogeneous devices for magnetic-field-based positioning, and attempted to solve this problem using a deep-learning approach.

Magnetic-field positioning can be divided into point-to-point matching and trajectory matching schemes. Recent works have attempted to implement point-to-point matching using deep-learning methods to classify magnetic-field measurements (magnetic landmarks) that had prominent features in indoor environments. Three LSTM-based DRNNs were proposed to classify magnetic-field landmarks by Bhattarai et al. in [34]. Magnetic landmarks were collected at 25 points in the corridor and 17 points in the laboratory, and the experimental results showed that this achieved 97.20% accuracy. Ashraf et al. [32] enabled three heterogeneous smartphones to collect various magnetic-field landmarks along indoor paths, transforming magnetic-field data into terms (words) and documents to mitigate the effects of smartphone heterogeneity. The extracted term frequency vectors were used to train LSTM and GRU networks, and their predictions were voted on to estimate the user’s current location.

Research into the classification of magnetic-field trajectories through deep learning has also been developed. In [28], the authors extracted recurrence-plot (RP), trend, sequence-length, and peak features from magnetic-field sequences. The extracted image features were then analyzed with a convolutional neural network (CNN), and magnetic landmarks were classified with a multilayer perceptron (MLP). The corridor and atrium had accuracies of 0.8 and 2.3 m, respectively. Zhang et al. [35] proposed an LSTM-based magnetic-field positioning algorithm and extended the magnetic-field dimension with a double sliding-window-based scheme, which expanded the feature dimension of the LSTM model to achieve higher positioning accuracy.

To solve the above problem, this paper proposes a novel magnetic positioning algorithm based on TCNs to avoid the RNN (LSTM and GRU) vanishing gradient problem. Magnetic-field sequences are used to represent each corridor [13]. A vast database of magnetic fields was collected through heterogeneous smartphones. We also designed a preprocessing system for the magnetic-field measurements to overcome the nature of heterogeneous devices, and improve algorithmic performance with the following consecutively stacked procedures: coordinate system transformation, moving average filtering, and first-order differencing.

## 3. Magnetic-Field Preliminaries

Magnetic-field measurements from heterogeneous devices are not the same [32,36], and the positioning accuracy can vary significantly when applying a positioning method to data from heterogeneous devices.

However, the magnetic-field measurements of heterogeneous devices on the same path show the same pattern [26], which is a good characteristic for magnetic indoor positioning.

It is labor- and time-intensive to create fine magnetic-field point maps. As the magnetic field varies between 25 and 65 μT, almost identical magnetic-field measurements may be repeated at different indoor locations, leading to low-magnetic-field-discernibility problems [37].

The magnetic trajectory model methodology is more reliable than point-based methods for magnetic-field positioning. It connects magnetic points in space to form a spatial sequence, inside which unique patterns can help in identifying specific areas and narrowing the positioning range.

**Figure 1:**
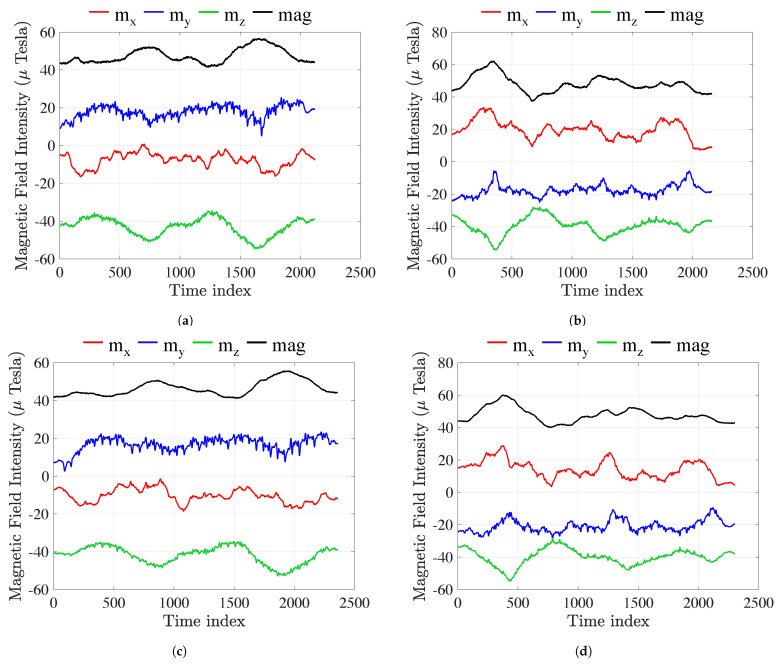

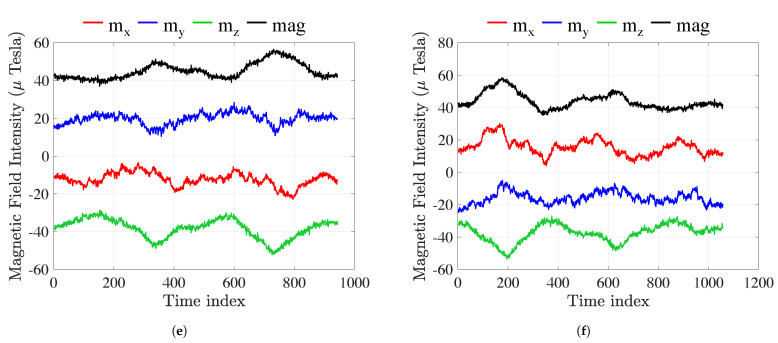
Magnetic field measurements of heterogeneous smartphones in the same corridor. (**a**) iPhone 12 Mini forward; (**b**) iPhone 12 Mini backward; (**c**) iPhone Xs Max forward; (**d**) iPhone Xs Max backward; (**e**) Redmi Note 7 forward; (**f**) Redmi Note 7 backward.

Figure 1 shows the $m_{x}$, $m_{y}$, and $m_{z}$ axes, and intensity components of the magnetic-field measurement of three heterogeneous smartphones. Figure 1a,c,e indicate trajectories in the forward direction along the corridor. Figure 1b,d,f indicate trajectories in the backward direction along the corridor, which are the inverse of Figure 1a,c,e. The magnetic trajectories of heterogeneous smartphones clearly exhibit similar patterns in the same corridor for all components. Symmetrical patterns can also be found between the forward and backward figures (e.g., Figure 1a,b).

Take Figure 1a,b as an example. The $m_{x}$ (red) and $m_{y}$ (blue) components of the magnetic field are direction-dependent, i.e., the magnetic declination equals $\arctan\frac{m_{y}}{m_{x}}$. It is difficult to see whether the correspondences of $m_{x}$ and $m_{y}$ were in opposite directions (forward and backward).

The value of $m_{z}$ is much larger than that of $m_{x}$ and $m_{y}$, contributing 90% of the intensity mag. Intuitively, $m_{z}$ and intensity mag show an axisymmetric relationship. We also used the information from $m_{x}$ and $m_{y}$ to improve the model’s robustness.

The magnetic-field trajectory’s spatial and temporal stability could help in finding the area where the user is located, such as the initial position of the PDR. The user walks with the smartphone with an arbitrary gesture and in an arbitrary direction, so it is essential to transform the raw magnetic-field measurement into a direction-independent coordinate.

The coordinate system of a smartphone, with the X axis pointing east, the Y axis pointing north, and the Z axis pointing to the sky, constitutes a right-handed coordinate system (ENU) [37]. The magnetic-field measurement is directional, mainly on the X and Y axes, while the Z axis reading is direction-independent. The calibrated magnetic-field intensity is constant at the same position [12].

The three-dimensional magnetic field can be decomposed into two horizontal and vertical components using the gravity vector, where the vertical component is parallel to the direction of gravity, and the horizontal component is orthogonal to gravity [38].

**Figure 2:**
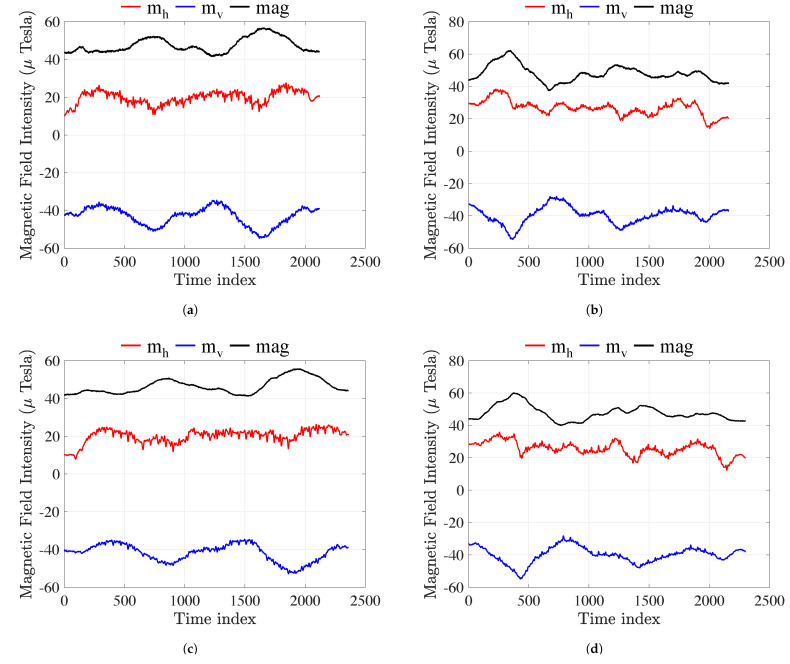

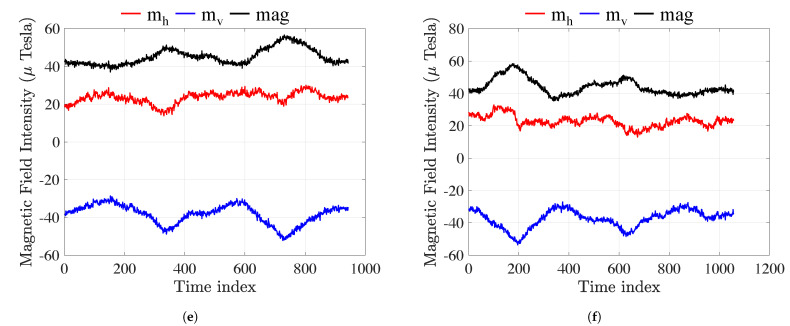
Magnetic field measurement with coordinate transformation in the same corridor. (**a**) iPhone 12 Mini transformed data forward; (**b**) iPhone 12 Mini transformed data backward; (**c**) iPhone Xs Max transformed data forward; (**d**) iPhone Xs Max transformed data backward; (**e**) Redmi Note 7 transformed forward; (**f**) Redmi Note 7 transformed data backward.

Figure 2 shows the transformation of the magnetic field into horizontal and vertical components. Figure 2a,c,e indicate transformed trajectories in the forward direction along the corridor. Figure 2b,d,f represents transformed trajectories in the backward direction along the corridor.

It was mentioned earlier that the $m_{x}$ and $m_{y}$ components are direction-dependent, while the $m_{z}$ and mag components are relatively stable. We, therefore, transformed the magnetic-field measurements into horizon vertical coordinates. Take Figure 2b as examples: the vertical component (blue) $m_{v}$ and the magnetic-field intensity mag (black) are centrosymmetric in the forward and backward directions, and since the horizontal component $m_{h}$ (red) is equal to $m_{h}=\sqrt{mag^{2}-m_{v}^{2}}$. We infer that $m_{h}$ is also centrosymmetric in the forward and backward directions.

## 4. Temporal Convolutional Networks

The temporal convolutional network is a class of neural network architecture with two distinctive characteristics: first, the convolution in the architecture is causal, meaning that future information does not influence previous information, and second, the input and output sequences have the same length [13].

### 4.1. Sequence Modeling

Suppose we have an input magnetic-field sequence $X=\left\{x_{1},x_{2}\ldots x_{T}|x_{i}\in R^{m}\right\}$ and wish to predict some corresponding outputs $Y=\left\{y_{1},y_{2}\ldots y_{T}\right\}$ (e.g., the labels of the corridor). We predict $y_{t}$ using only previously observed inputs: $X=\left\{x_{1},x_{2}\ldots x_{t}|x_{i}\in R^{m}\right\}$. A magnetic-field sequence modeling network can be expressed as a function $f:X^{t+1}\to Y^{t+1}$, namely:

(1) $$\begin{array}{c} {\hat{y}}_{0},\ldots ,{\hat{y}}_{t}=f\left(x_{0},\ldots ,x_{t}\right). \end{array}$$

The restriction on $y_{t}$ is dependent only on $\left\{x_{1},x_{2}\ldots x_{t}\right\}$, and not on any "future" input $\left\{x_{t+1},x_{t+2}\ldots x_{T}\right\}$. Our goal is to find a network *f* that minimizes the expected loss between actual and predicted values, $L\left(y_{0},\ldots ,y_{T},f\left(x_{0},\ldots ,x_{T}\right)\right)$.

### 4.2. Causal Convolutions

RNNs are often used for sequence modeling, such as processing video, audio, and sensor signals along the time direction, and CNNs are often used for image processing. However, CNNs are significantly underestimated for sequential modeling and build more concise models than RNNs do.

(2) $$\begin{array}{c} p\left(x\right)=\prod_{t=1}^{T}p\left(x_{t}|x_{1},\ldots ,x_{t-1}\right) \end{array}$$

Conventional 2D CNN models are not designed for dealing directly with sequence data, but 1D causal convolutions can perform sequence modeling, mainly abstracting to predict $y_{t}$ on the basis of $\left\{x_{1},x_{2}\ldots x_{t}\right\}$ and $\left\{y_{1},y_{2}\ldots y_{t-1}\right\}$, making $y_{t}$ close to the actual value.

**Figure 3:**
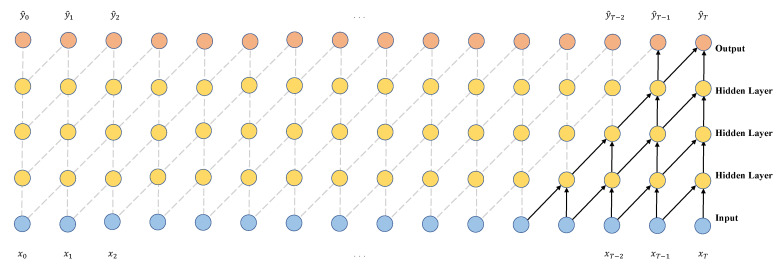
Visualization of a stack of causal convolutional layers.

Causal convolution requires many layers or large filters to increase the receptive fields of the convolution. As shown in Figure 3, an output corresponds to more inputs when many hidden layers exist between the output and input layers. The more the hidden layers between the input and output layers, the farther apart they are, and the higher the convolutional computation is, which can bring problems such as gradient vanishing, high training complexity, and poor fitting.

### 4.3. Dilated Causal Convolutions

Dilated convolution can be applied to regions larger than the length of the filter by skipping some of the input, and it is equivalent to generating a larger filter from the original filter by adding zeros.

**Figure 4:**
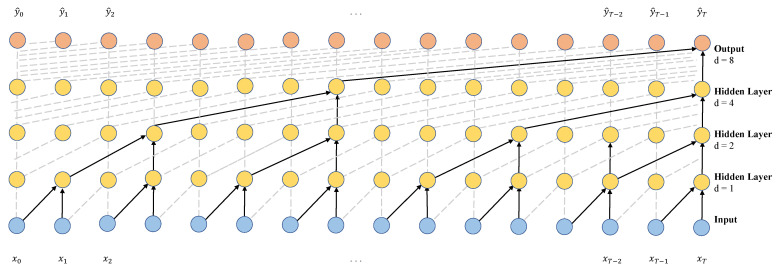
Visualization of a stack of dilated causal convolutional layers.

Suppose that a network has *N* convolutional layers, the dilated factor of the *n*-th convolutional layer is 2(n−1), the span is 1, and the filter size is $f_{size}$; then, the receptive field size of the network can be computed as $R=\left(f_{size}-1\right)\left(2^{N}-1\right)+1$. Figure 4 shows the dilated causal convolutions of 1, 2, 4, and 8.

The size of the receptive field and the number of learnable parameters can be adjusted by changing the filter’s size and the number of layers. Dilated convolution allows for a model to have a very large receptive field with a small number of layers, which can solve the problems associated with causal convolution [13,39,40].

### 4.4. Residual Block

A deep neural network can be viewed as mapping between the input and output spaces. It is composed of multiple stacked layers. Each layer is a subfunction regarding its underlying mapping.

Deep neural networks face the problem of degradation, and researchers have found that, as the depth of the network increases, the accuracy becomes saturated and degrades rapidly.

**Figure 5:**
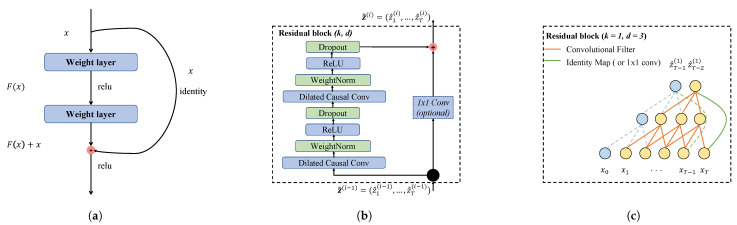
Structure of proposed temporal convolutional networks. (**a**) Residual learning block; (**b**) TCN residual block; (**c**) example of residual connection in a TCN.

He et al. [41] proposed deep residual learning to solve this degradation problem. Figure 5a depicts the residual learning block. Assume that H(x) is an underlying mapping composed of multiple stacked layers, with x representing the input of the initial layer. The residual mapping is represented as F(x)=H(x)−x

It is challenging to approximate identity mapping by directly using multiple nonlinear layers. If the identity mapping is optimal, residual learning reconstruction approximates the identity mapping by reducing the weights of multiple nonlinear layers to zero.

Since the receptive field of a TCN is determined via network depth *n*, filter size *k*, and dilation factor *d*, Bai et al. [13] designed a generic TCN model that solved the recession problem for deeper and larger TCNs by replacing the convolutional layers with a generic residual module.

Figure 5b depicts the residual blocks of a generic TCN architecture, including two sets of dilated causal convolution layers with the same dilation factor, weight normalization, rectified linear unit (ReLU) activation function, and spatial dropout.

Figure 5c The TCN network combines the input and output of each block, and when the input dimension does not equal the output dimension, an additional 1 × 1 convolution is performed on the input to ensure dimensional matching.

### 4.5. Advantages and Disadvantages

There are several advantages and disadvantages to TCN sequence modeling [13]. Its advantages are listed as follows.

- *Parallelism*: RNNs process time sequences sequentially and must wait for the completion of the preceding sequence before performing predictions for the subsequent sequence. Since convolution enables the use of the same filter at each layer, TCN allows for the input sequence to be treated as a whole.
- *Flexible receptive field size*: To modify the size of receptive fields, TCNs can stack more dilation (causal) convolutional layers, employ larger dilation factors, or increase the size of the filters.
- *Stable gradients*: Since the backpropagation path of TCN is different from the temporal direction of the sequence, it avoids the explosion/gradient disappearance problem of RNNs (LSTM, GRU).
- *Low memory requirement for training*: Training requires less memory for TCNs. In TCNs, cell gates are shared within a layer, and the backpropagation path depends exclusively on network depth. LSTM and GRU typically require a substantial amount of memory to store the partial outcomes of their numerous cell gates.
- *Arbitrary length input*: TCNs obtain sequences of arbitrary length by sliding one-dimensional convolutional kernels, while RNNs simulate input sequences of different lengths by recursion.

TCN also has a distinct disadvantage.

- *Insufficient flexibility in transfer learning*: TCN may not be as transferable because the amount of historical information necessary for model prediction may vary across domains. As a result, the performance of TCNs may be poor when transferring a model from a problem that requires less memory information to a problem that requires more memory, as their receptive field is insufficiently large.

## 5. Experiments

In this section, we outline a framework we designed for a magnetic-field indoor positioning system on the basis of TCNs. Numerous magnetic-field trajectories were collected in an indoor corridor using a heterogeneous smartphone, and the magnetic-field data were preprocessed via coordinate transformation, moving average, and first-order differencing. Trained and untrained smartphones were used to evaluate the algorithm.

### 5.1. System Architecture

**Figure 6:**
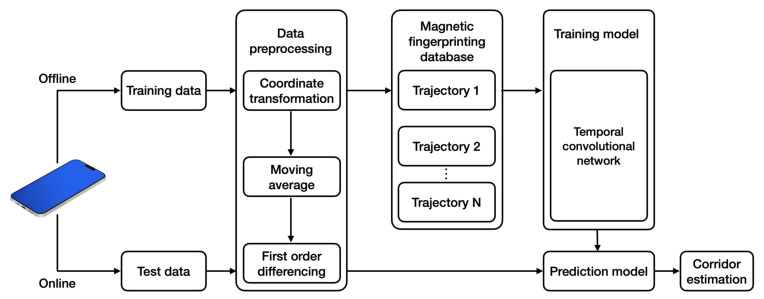
Scheme of the indoor magnetic trajectory classification based on a temporal convolutional network.

Figure 6 depicts the framework of the proposed TCN-based magnetic trajectory classification system. The system comprises two phases: offline training and online test. A smartphone equipped with a magnetometer was used to classify magnetic trajectories as follows:

- The magnetic-field database was collected from the building’s corridors.
- Coordinate system transformation, smoothing filtering, and first-order differencing were implemented to obtain magnetic-field features.
- The preprocessed magnetic-field measurements were combined to build a training set of magnetic fingerprinting for each corridor with the corridor number as the label.
- The collected database of magnetic-field trajectories was used to train the TCN model.
- The test dataset was used to evaluate the trained prediction model.

### 5.2. Data Collection

**Figure 7:**
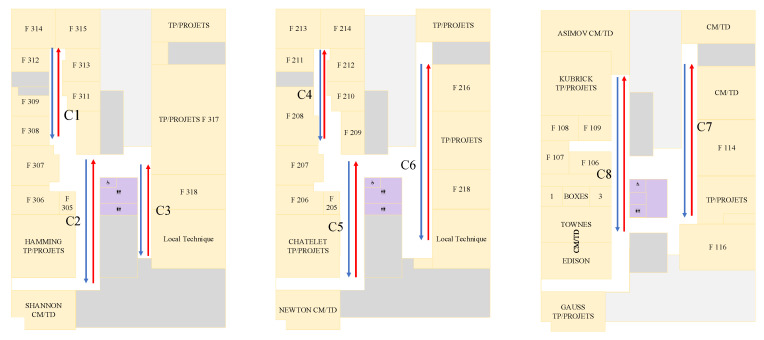
Designed corridors in Polytech Galilee.

To evaluate the performance of the proposed algorithm, we selected eight corridors on the first, second, and third floors of the building of Polytech Galilée, shown in Figure 7; several heterogeneous smartphones (iPhone Xs Max, iPhone 12 Mini, Redmi Note 7, Samsung Galaxy S20) were used to collect the data.

Table 1 describes the system version, sensor vendor, magnetometer model, and magnetometer characteristics of the smartphones used in the experiment. Samsung Galaxy S20, Samsung Galaxy S9, and Redmi Note 7 all use magnetometer models from Asahi Kasei Microdevices (AKM), OnePlus 7T Pro uses a magnetometer model from MEMSIC, while iPhone uses Apple’s own magnetometer, and the information is not available through the API.

The chosen corridors were all between 10 and 20 m in length, the MATLAB Mobile application was used for data collection, and the sampling frequency was set to 100 Hz. The smartphones were held horizontally, and data were collected 10 times in the forward direction and 10 times in the backward direction, so that there were 20 trajectories per corridor; the training dataset of 3 heterogeneous smartphones (iPhone Xs Max, iPhone 12 Mini, and Redmi Note 7) contained a total of 3×10×2×8=480 trajectories. We then collected two more round trips in each corridor (two forward and two backward) as a test dataset, giving a total of 4×8×3=96 test trajectories. The training dataset had 739,700 magnetic-field measurement samples, while the test dataset contained 148,600 samples.

We also took two round-trip paths of Samsung Galaxy S20, Samsung Galaxy S9, and OnePlus 7T Pro to test whether the algorithm could be seamlessly applied to heterogeneous smartphones, even if we had not used the neural network to train them.

### 5.3. Magnetic Features Preprocessing

The magnetic-field sequence underwent three preprocessing steps: coordinate system transformation, smoothing filtering, and first-order differentiation.

- *Coordinate transformation*: The original magnetic-field signal needed to be transformed from a body coordinate system into a world coordinate system.
  (3) $$\begin{array}{c} m_{t}^{n}=R_{t}^{nb}m_{t}^{b} \end{array}$$
  where $m_{t}^{b}=\left(m_{x,t}^{b},m_{y,t}^{b},m_{z,t}^{b}\right)\in R^{3\times 1}$ is the magnetic-field measurement in the body coordinate system at time *t*, and $m_{t}^{n}=\left(0,m_{h,t}^{n},m_{v,t}^{n}\right)\in R^{3\times 1}$ is the magnetic-field measurement in the world coordinate system at time *t*, $R_{t}^{nb}\in R^{3\times 3}$ is the rotation matrix that transforms the magnetic-field measurement from the body coordinate system *b* to the world coordinate system *n*.
  After the coordinate transformation, we used the magnetic-field horizontal component, vertical component, and the magnetic-field intensity as features: $m=(m_{h},m_{v},\sqrt{m_{h}^{2}+m_{v}^{2}})$.
- *Smoothing filter*: As the collected magnetic-field sequence contained Gaussian white noise and burrs, we employed the moving average approach with a window size of 100 to smooth the signal.
- *First order difference*: After transforming the coordinate system and smoothing filter, we calculated the difference between adjacent elements of the magnetic-field sequence as features.

### 5.4. Experimental Settings

Table 2 describes the parameter settings of the algorithm. We defined a TCN network with six residual blocks in sequence, beginning with a dilation factor of 1 and each subsequent residual block with a dilation factor twice that of the previous layer. For the residual block’s one-dimensional convolutional layer, 128 filters of size 5 were provided, and a dropout factor of $5\times 10^{-3}$ was specified for the dropout layer. The optimizer was set to ‘*Adam*’, epochs were set to 120, the minibatch size was set to 4, and the learning rate was set to $1\times 10^{-4}$.

Table 2 also shows the working environment of the experiment. The experiments in this article were conducted on a MacBook Pro with a 2.6 GHz 6-Core Intel Core i7 processor running macOS Monterey 12.6. All models were implemented on MATLAB 2022a.

### 5.5. Classification Results

**Figure 8:**
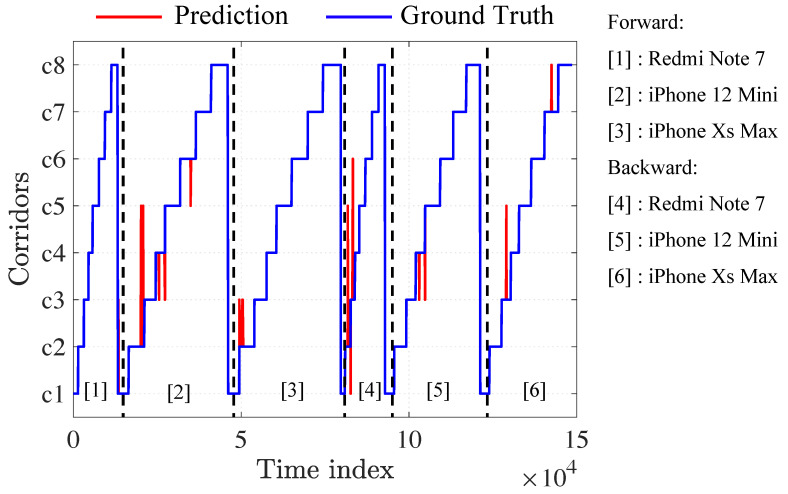
Test trajectory predictions.

We first tested our prediction model using the three trained smartphones. The test dataset for the experiment consisted of forward and backward trajectories from the three trained smartphones. Figure 8 depicts the ground truth and predictions of the test set, with most of the red lines overlapping the blue (predictions were consistent with the ground truth). The shown red line represents an incorrect prediction.

**Figure 9:**
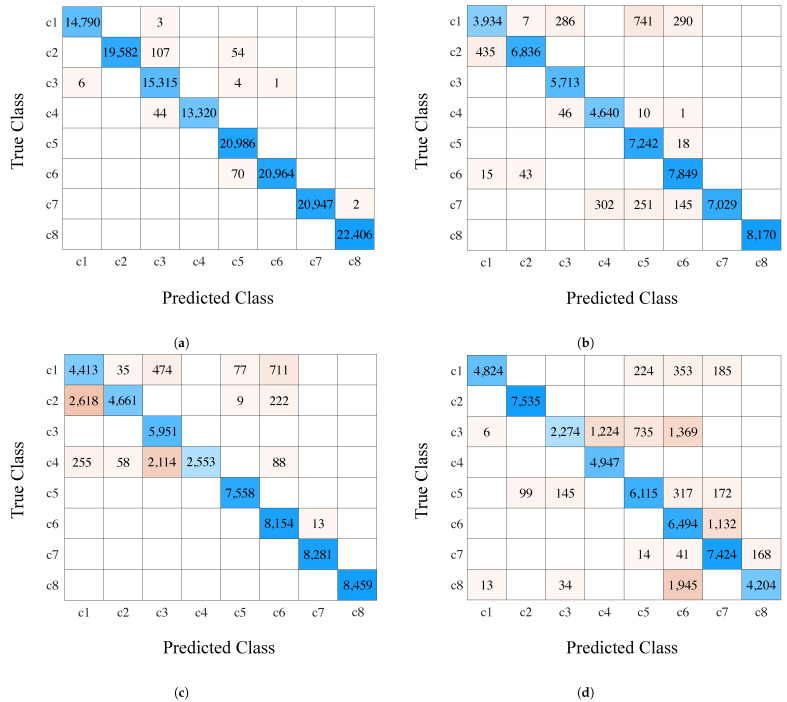
Confusion matrix trained and untrained smartphone. (**a**) Aggregation of three trained smartphones (accuracy: 99.80%); (**b**) Samsung Galaxy S20 (accuracy: 95.20%); (**c**) Samsung Galaxy S9 (accuracy: 88.23%); (**d**) OnePlus 7T Pro (accuracy: 84.27%).

Figure 9a shows the confusion matrix result of the three trained smartphones (iPhone Xs Max, iPhone 12 Mini, and Redmi Note 7). The blue diagonal areas represent correctly predicted points, while the nondiagonal parts represent wrongly predicted points. The majority of the 148,600 total points corresponded to correct predictions. Classification accuracy can be evaluated by comparing predictions with ground truth and calculated with Equation (4); the accuracy of the TCN-based magnetic-field trajectory classification method was 99.80%.

(4) $$\mathrm{Accuracy}=\frac{\#\;\mathrm{correctly}\;\mathrm{classified}\;\mathrm{points}}{\#\;\mathrm{total}\;\mathrm{points}}.$$

To evaluate the applicability of our trained model to an untrained smartphone, we utilized a Samsung Galaxy S20, Samsung Galaxy S9, and OnePlus 7T Pro to collect three test datasets (two round-trip walks in eight corridors for each smartphone). The newly collected data were fed into the previously trained model (Figure 9b–d) and achieved accuracies of 95.20%, 88.23%, and 84.27%, respectively. This demonstrates that the trained model could also be applied to untrained smartphones.

We implemented the GRU and bidirectional LSTM (BiLSTM) to the same training and test sets. Table 3 compares the classification accuracy of BiLSTM, GRU, and TCNs in the same dataset. The results show that the TCN models outperformed the two RNN models.

## 6. Conclusions

In this article, we proposed a novel TCN-based indoor magnetic positioning algorithm for smartphones that exploits the predictive power of TCNs to solve the indoor magnetic positioning problem and avoids the time-consuming fingerprint matching process compared to the DTW-based magnetic-field sequence matching method. Compared with traditional RNN methods such as LSTM and GRU, our training was faster and more accurate, and avoided the gradient explosion problem.

We analyzed the characteristics of magnetic-field trajectories, and preprocessed the magnetic-field sequence using coordinate transformation, smoothing filters, and first-order differencing. Large-scale magnetic-field trajectory data were used to train the prediction model, and different test sets were used to evaluate our algorithm. An accuracy of 99.8% for the three trained smartphones was achieved. Accuracies of 95.20%, 88.23%, and 84.27% were achieved for the three untrained heterogenous smartphones. In addition, the TCN algorithm was significantly more efficient than models from GRU and BiLSTM.

## Figures and Tables

**Table 1:** Magnetometer information and operating systems for heterogeneous smartphones.

Smartphone	Operating System	Magnetometer	Sensor Vendor	Description
Samsung Galaxy S20	Android 11	AK09918C	AKM	3-axis, 16-bit; Sensitivity: 0.15 μT/LSB
Samsung Galaxy 9	Android 9.0	AK09916C	AKM	3-axis, 16-bit; Sensitivity: 0.15 μT/LSB
OnePlus 7T Pro	Android 11	MMC5603X	MEMSIC	3-axis, 16-bit; Sensitivity: 0.15 μT/LSB
Redmi Note 7	Android 10	AK09918	AKM	3-axis, 16-bit; Sensitivity: 0.15 μT/LSB
iPhone Xs Max	iOS 15.61	∼	∼	∼
iPhone 12 Mini	iOS 16.0.2	∼	∼	∼

**Table 2:** TCN parameterization and experimental environment.

Parameter	
Number of filters	128
Filter size	5
Dropout factor	$5\times 10^{-3}$
Number of blocks	6
Optimizer	Adam
Epochs	120
Minibatch size	4
Learning rate	$1\times 10^{-4}$
Operating system	macOS Monterey 12.6
CPU	2.6 GHz 6-Core Intel Core i7
Platform	MATLAB 2022a

**Table 3:** Prediction accuracy with the trained and untrained smartphones.

Models	Trained Smartphones	Galaxy S20	Galaxy S9	OnePlus 7T Pro
BiLSTM	85.51%	36.36%	60.53%	62.23%
GRU	76.97%	34.34%	43.36%	43.61%
TCN	99.80%	95.20%	88.23%	84.27%

## Data Availability

Not applicable.
